# Rapid morphological change in UK populations of *Impatiens glandulifera*

**DOI:** 10.1038/s41598-024-69710-y

**Published:** 2024-08-20

**Authors:** A. L. Wyatt, H. S. Pardoe, C. J. Cleal, J. Sánchez Vilas

**Affiliations:** 1 Geobiology and Geochemistry Division, Cardiff School of Earth and Environmental Sciences, Cardiff University, Main Building, Park Place, Cardiff, CF10 3AT UK; 2 Department of Natural Sciences, Amgueddfa Cymru - Museum Wales, Cathays Park, Cardiff, CF10 3NP UK; 3 School of Earth Sciences, University of Bristol, Bristol, BS8 1TQ UK; 4 Organisms and Environment Division, Cardiff School of Biosciences, Cardiff University, The Sir Martin Evans Building, Museum Avenue, Cardiff, CF10 3AX UK; 5 Departamento de Bioloxía Funcional (Área de Ecoloxía), Facultade de Bioloxía, Universidade de Santiago de Compostela, c/ Lope Gómez de Marzoa s/n, 15782 Santiago de Compostela, Spain

**Keywords:** Ecology, Plant sciences

## Abstract

The highly invasive *Impatiens glandulifera* (Himalayan balsam) is one of the most prolific and widespread invasive plants in the British Isles. Introduced in the early nineteenth century, it has now been reported in almost every vice county across the UK and is a fierce competitor that has adverse effects on the local community structure. Despite the negative impacts that invaders like *I. glandulifera* have on local communities, there have been very few studies which address the morphological changes that invasive plant populations have undergone since their initial introduction. This is the first study of its kind to investigate the morphological changes that have occurred in *I. glandulifera*. 315 herbarium specimens dating from 1865 to 2017 were used to measure changes in morphological traits such as leaf size, flower length and stomatal characteristics. We found that since 1865, there has been a significant reduction in overall leaf size, a significant reduction in stomatal density and a significant increase in the overall flower length. These results highlight the importance of monitoring the evolutionary change in prolific alien species over the course of their invasion, providing useful insights into changes in competitive ability which may prove useful in managing dispersal and providing options for potential management.

## Introduction

In the last 100 years, globalization and industrialisation have exponentially increased the incidence of invasions by non-native plants^1^. There is increasing concern over the resulting harm to the environment; invasive species are now regarded as a leading cause of plant extinction, second only to habitat loss^2–4^. Species introduced to new regions may face very different environmental conditions to those experienced in their native range, and there is evidence that many successful invasive species seem to undergo rapid phenotypic changes in response to the challenges posed by the novel/new environment^5,6^.

These rapid phenotypic changes may be the result of evolution^5^. Reproductively isolated from their source populations and faced with novel selection pressures, introduced populations must adapt quickly to their new environment to establish a stable population in their new range. Introduced populations often have a low propagule size and low genetic diversity^7,8^, which would usually reduce their evolutionary potential^9^. However, genetic bottlenecks in such invasive species can restrict gene flow^10^ causing a rapid divergence of the phenotypes from their native range. Individuals that are better adapted to the new environment can appear rapidly and out-compete more poorly-adapted individuals during the lag phase of the invasion^11,12^, with adaptive traits evolving in 20 generations or less^13^. Moreover, a lack of co-evolved parasites and herbivores means non-native species may not need some costly defence strategies (e.g. secondary chemical production). This allows for resources to be invested in other traits advantageous to their new range, which will increase their competitive ability and facilitate implantation in new habitats and, consequently, their expansion into new areas^14–16^.

Rapid phenotypic change may also be attributed to phenotypic plasticity, resulting from genotypes producing different phenotypes in response to different environmental conditions^17^. High phenotypic plasticity is a common trait in invasive plant species, and has been demonstrated in the Himalayan balsam (*Impatiens glandulifera* Royle)^18^. However, regardless of whether it is caused by evolution or phenotypic plasticity, rapid phenotypic changes in response to novel environmental conditions could play a role in facilitating the spread of invasive plants^15^. Previous research that investigated phenotypic change in invasive species in response to novel environments were based on comparisons between native and introduced populations of the same species^11,19^. Very few studies have considered the magnitude and direction of change through time since first invasion.

The present study will focus on *I. glandulifera,* a species native to the Himalayas that was first introduced into the UK in 1839 as a garden ornamental. It has now become a widespread and problematic invasive species found throughout most of Europe, as well as in North America, Russia, Canada and New Zealand^20–23^. *I. glandulifera* is a summer annual herb that reproduces only by seed and is typically found in riparian habitats in the UK. Germination takes place between February and March, with flowering occurring from July to October. The plants begin setting seed from mid-July and die back by the end of autumn with the first hard frosts^21,24^. A fierce competitor, *I. glandulifera* reaches up to 2.5 m tall in the UK, has a fast growth rate, over-produces nectar, and produces up to 2500 seeds per plant^21,22,25^. Forming dense monospecific stands with foliage that swamps non-native plant species, *I. glandulifera* can reduce species richness in invaded habitats by 25%^26^, releasing allelopathic chemicals into the environment^27^ and promoting soil erosion in riparian habitats^28^. Effective management of *I. glandulifera* populations in the UK requires preventing flowering for several years to deplete the seed bank. Physical (hand-pulling, mowing), chemical (herbicides) and biological methods (rust fungus, *Puccinia komarovii* var. *glanduliferae*)^29^ of control are being used in the UK, with management costs estimated at approximately £1 million per annum UK-wide^23^.

In Britain, it grows taller, has higher fecundity and a larger total leaf area than in its native range, making the non-native populations more competitive than their native counterparts^30^. In this study, we used herbarium specimens to investigate changes in populations of *I. glandulifera* since its introduction into the U.K. This study looked at features which would give *I. glandulifera* an adaptive advantage, including leaf length, leaf width, leaf area, stomatal density and flower length.

## Results

### Leaf area, length and width

**Figure 1:**
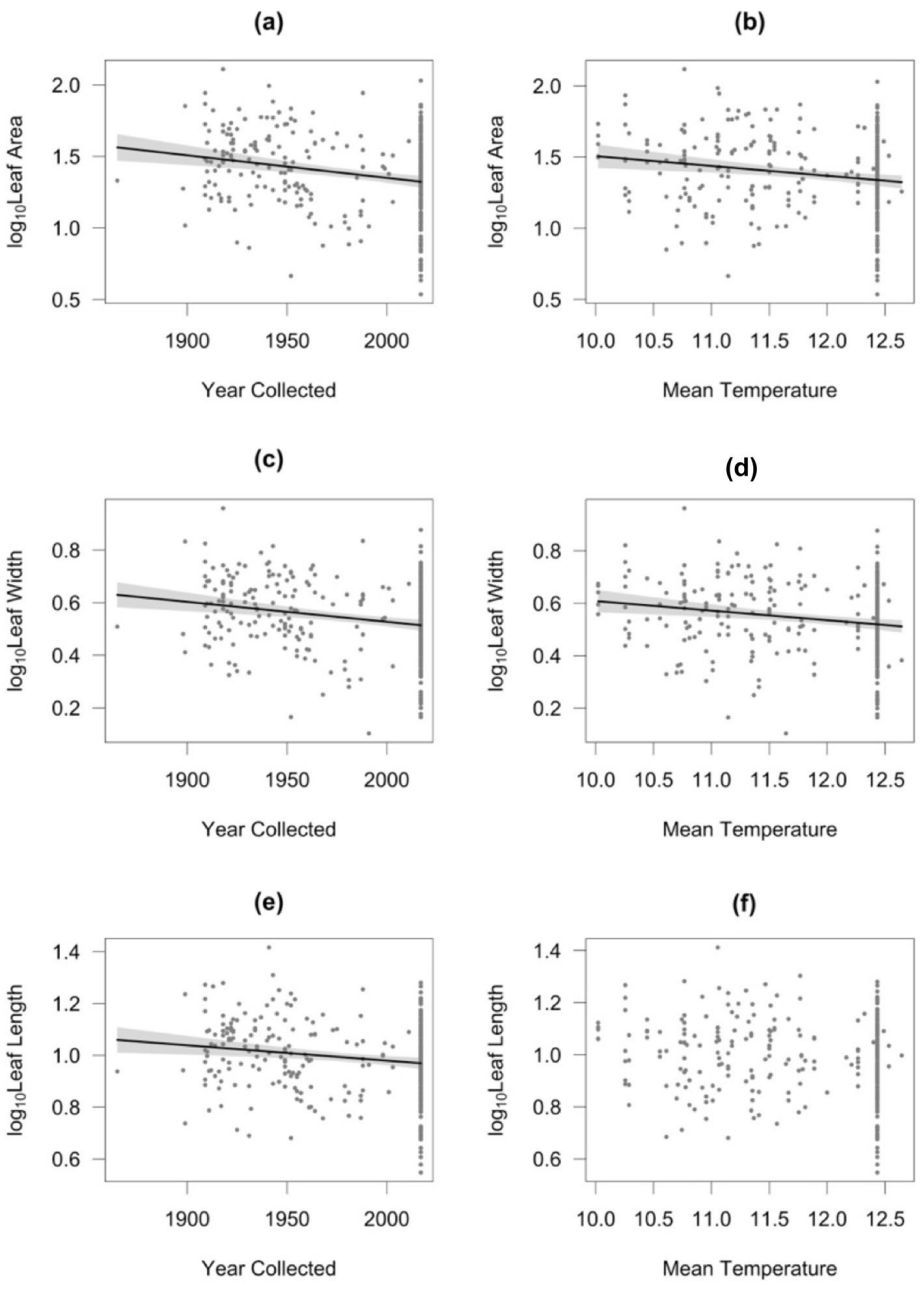
Partial regression plots of the effects of date of collection (Year Collected) and Mean Temperature (mean of average monthly temperatures from March to November) on Leaf Area (**a** and **b**, respectively), Leaf Width (**c** and **d**, respectively) and Leaf Length (**e** and **f**, respectively). Lines represent model predictions of statistically supported effects; grey bands indicate 95% confidence bands. The adjusted R^2^, and *P*-values for the fitted models are given in Table 1.

Leaf area, leaf width and leaf length decreased with increasing time (*P* < 0.01; Table 1). In particular, when comparing the first four decades sampled (1860s, 1890s, 1900s, and 1910s) with the last four decades (1980s, 1990s, 2000s, and 2010s), leaf area decreased from 36.7 ± 4.3 cm^2^ to 25.9 ± 1.2 cm^2^, leaf width decreased from 4.20 ± 0.24 cm to 3.48 ± 0.08 cm and leaf length decreased from 11.13 ± 0.61 cm to 9.84 ± 0.24 cm. In addition, leaf area and leaf width decreased also as a function of increasing mean temperature of the growing season (*P* < 0.05, Table 1, Fig. 1). However, the effects of the leaf traits in response to the mean temperature of the growing season need to be treated with caution, as the percentage of models (ran after under-sampling the last decade to N = 24) was low particularly for leaf area and leaf length. No significant effect of geographical location (as indicated by Latitude and Longitude) was found on leaf area, leaf width or leaf length (see Table 1).

**Table 1:** Estimated regression parameters, standard errors, t-values and *P*-values for the models performed on the leaf traits measured in *I. glandulifera*.

Variables and fitted models	Estimate	Standard error	t	*P*-value	Adjusted R^2^	Percentage of models with *P* < 0.05
**Log**_**10**_** leaf area**						
*Model 1*				< *0.001*	*0.048*	
Intercept	5.6500	1.2464	4.533	** < 0.001**		*100%*
Year Collected	− 0.0016	0.0004	− 4.112	** < 0.001**		97%
Latitude	− 0.0220	0.0177	− 1.239	0.216		0%
Longitude	0.0026	0.0140	0.185	0.854		0%
*Model 2*				*0.029*	*0.007*	
Intercept	3.1259	0.9701	3.222	**0.001**		100%
Mean Temperature (March–Nov)	− 0.0691	0.0215	− 3.216	**0.001**		45%
Latitude	− 0.0176	0.0178	− 0.988	0.324		0%
Longitude	0.0065	0.0140	0.465	0.642		0%
**Log**_**10**_** leaf width**						
*Model 1*				*0.002*	*0.038*	
Intercept	2.4705	0.6388	3.867	** < 0.001**		100%
Year Collected	− 0.0008	0.0002	− 3.866	** < 0.001**		95%
Latitude	− 0.0085	0.0091	− 0.934	0.351		0%
Longitude	− 0.0057	0.0072	− 0.795	0.427		0%
*Model 2*				*0.062*	*0.011*	
Intercept	1.2989	0.4951	2.623	**0.009**		100%
Mean Temp (March–Nov)	− 0.0365	0.0110	− 3.328	**0.001**		68%
Latitude	− 0.0065	0.0091	− 0.719	0.473		0%
Longitude	− 0.0042	0.0072	− 0.589	0.556		0%
**Log**_**10**_** leaf length**						
*Model 1*				*0.008*	*0.028*	
(Intercept)	2.6614	0.6552	4.062	** < 0.001**		100%
Year Collected	− 0.0006	0.0002	− 2.934	**0.004**		73%
Latitude	− 0.0092	0.0093	− 0.989	0.324		0%
Longitude	0.0073	0.0073	0.995	0.320		2%
*Model 2*				*0.015*	*0.049*	
(Intercept)	1.6866	0.5081	3.319	**0.001**		100%
Mean Temp (March–Nov)	− 0.0239	0.0113	− 2.125	**0.034**		12%
Latitude	− 0.0075	0.0093	− 0.801	0.423		0%
Longitude	0.0091	0.0073	1.233	0.219		4%

*P*-values for the estimated regression parameters are marked in bold to denote statistical significance at *P* < 0.05. The adjusted R^2^, *P*-values for the fitted models are given in italics. Percentage of the models with *P* < 0.05 run on the dataset after 1000 iterations of random under-sampling to limit the last decade to n = 24.

### Stomatal density

**Figure 2:**
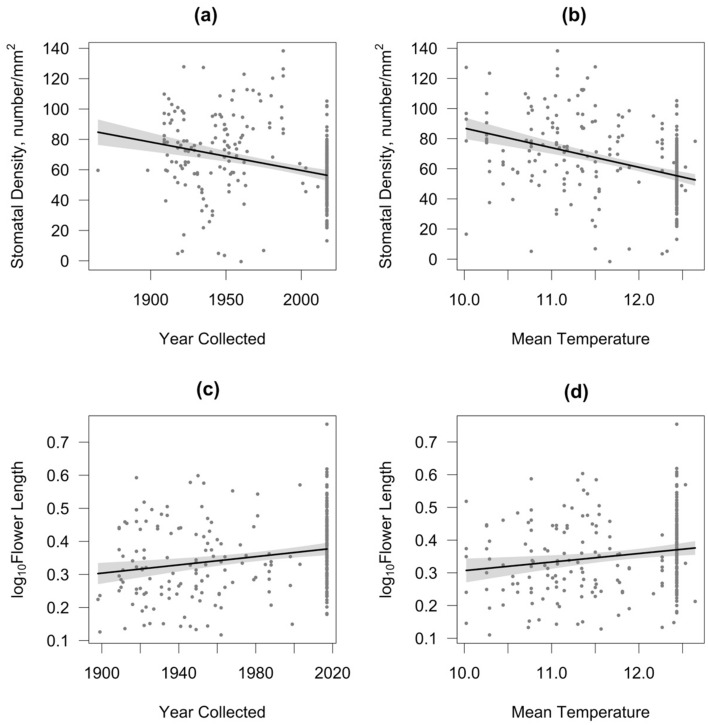
Partial regression plots of the effects of date of collection (Year Collected) and Mean Temperature (mean of average monthly temperatures from March to November) on Stomatal Density (**a** and **b** respectively) and on Flower Length (**c** and **d** respectively). Lines represent model predictions of statistically supported effects; grey bands indicate 95% confidence bands. The adjusted R^2^, and *P*-values for the fitted models are given in Table 2.

Stomatal density decreased significantly with increasing time (*P* < 0.001, Table 2), from 76 ± 4 stomata/mm^2^ in the first four decades sampled (1860s, 1890s, 1900s and 1910s) to 57 ± 2 stomata/mm^2^ in the last four decades decades (1980s, 1990s, 2000s and 2010s). However, this result was not supported when re-running the models after under-sampling the original dataset (Table 2, Fig. 2a). Stomatal density decreased significantly with increasing mean temperature across the growing season (*P* < 0.001, Table 2, Fig. 2b). No significant effect of geographical location (as indicated by Latitude and Longitude) was found on stomatal density (see Table 2).

**Table 2:** Estimated regression parameters, standard errors, t-values and *P*-values for the models performed on stomatal density and flower length (cm) in *I. glandulifera*.

Variables and Fitted models	Estimate	Std. Error	t	*P*-value	Adjusted R^2^	Percentage of models with *P* < 0.05
**Stomatal density (number/mm**^**2**^**)**						
*Model 1*				< *0.001*	*0.088*	*10.44*
(Intercept)	421.3571	119.2098	3.535	** < 0.001**		1%
Year Collected	− 0.1866	0.0342	− 5.458	** < 0.001**		5%
Latitude	0.2038	1.7841	0.114	0.909		0%
Longitude	− 0.2994	1.2613	− 0.237	0.812		0%
*Model 2*				< *0.001*	*0.146*	
(Intercept)	184.3143	92.1843	1.999	**0.047**		0%
Mean Temp (March–Nov)	− 12.9903	1.8021	− 7.208	** < 0.001**		100%
Latitude	0.6123	1.7190	0.356	0.722		0%
Longitude	− 0.3233	1.2076	− 0.268	0.789		0%
**Flower length (cm)**						
*Model 1*				*0.005*	*0.039*	
(Intercept)	− 1.0068	0.5649	− 1.782	0.076		3%
Year Collected	0.0006	0.0002	3.518	** < 0.001**		53%
Latitude	0.0024	0.0079	0.303	0.762		0%
Longitude	− 0.0002	0.0061	− 0.035	0.972		0%
*Model 2*				*0.040*	*0.021*	
(Intercept)	− 8.20 × 10^–5^	4.33 × 10^–1^	0.000	0.999		0%
Mean Temp (March–Nov)	2.62 × 10^–2^	9.53 × 10^–3^	2.748	**0.006**		3%
Latitude	7.50 × 10^–4^	7.97 × 10^–3^	0.094	0.925		0%
Longitude	− 2.08 × 10^–3^	6.10 × 10^–3^	− 0.343	0.732		0%

*P*-values for the estimated regression parameters are marked in bold to denote statistical significance at *P* < 0.05. The adjusted R^2^, and *P*-values for the fitted models are given in italics. Percentage of the models with *P* < 0.05 run on the dataset after 1000 iterations of random under-sampling to limit the last decade to n = 24.

### Flower length

Flower length increased significantly with increasing time (*P* < 0.001, Table 2, Fig. 2c), from 2.20 ± 0.12 cm in the first four decades sampled (1860s, 1890s, 1900s, and 1910s) to 2.45 ± 0.05 cm in the last four decades (1980s, 1990s, 2000s, and 2010s). Flower length also increased with increasing mean temperature of the growing season (*P* = 0.006, Table 2, Fig. 2c, d). Here, again, one needs to be cautious interpreting these results, particularly with regards to changes with increasing mean temperature, as only 3% of the models re-ran after under-sampling rendered *P*-values < 0.05 (Table 2). No significant effect of geographical location (as indicated by Latitude and Longitude) was found on flower length (see Table 2).

## Discussion

Results of the present study indicate distinct morphological changes over time in all traits measured in UK populations of *I. glandulifera*: leaf area, leaf length, leaf width and stomatal density have all decreased since 1865, whilst flower length increased since 1865. This suggests that there have been microevolutionary changes in UK populations of *I. glandulifera,* with a shift towards less competitive leaf traits, but an increase in flower length. This latter trend may reflect directional selection driven by pollinators.

### Leaf length, width and area

According to the Evolution of Increased Competitive Ability hypothesis (EICA) traits such as increased height, leaf area and growth rate that give a non-native species a competitive advantage appear early during biological invasions and can result in aggressive phenotypes^31,32^. However, there have been inconsistent results in the literature on leaf characteristics in invasive species: some studies suggest that leaf area increased after the initial invasion^11,33,34^ but an increasing number of studies have reported a reduction in total leaf area after invasion^35–37^. The results of the present study on *I. glandulifera* support the latter view, with leaf area, length and width all decreasing since 1865 (Fig. 1). Our study also found that leaf area and leaf width decreased with increasing mean temperature, a trend suggested by other studies^38^. A milder climate in the UK may explain the larger leaf area of *I. glandulifera* in the UK compared to the native range^30^. However, we might expect a decrease in leaf area associated with higher temperatures as found in this study^39^. Smaller leaves appear to have better thermal regulation than larger leaves^40^, but regulation of leaf size is complex and can also be strongly influenced by other components of climate, such as precipitation^41^.

Although not measured in this study, a greater investment in the number of leaves produced as a result of an increase in height, could explain a reduction in leaf size as proposed by the leaf size-number trade-off theory^42^. Indeed, in the introduced range, *I. glandulifera* has been found to be taller than plants in the native range^30,43,44^. Several advantages have been suggested for an increase in leafing intensity, including a greater potential for higher fecundity allocation as a result of an increase in lateral inflorescences^42^. In fact, taller plants in *I. glandulifera* have been found to produce more seeds in UK populations^45^. In addition, taller plants may also benefit from an advantage in the competition for light^46^.

### Stomatal density

Although decreases in stomatal density with time are consistent with previous findings in the literature and have been linked to the sensitivity of this trait in response to increasing CO_2_ levels^47–49^, we must be cautious interpreting this result as it lacks support after re-running the models from datasets obtained after random re-sampling of the last decade. Decreases in stomatal density have also been reported in response to increasing temperatures, as a possible mechanism to help reduce water loss by reducing the stomatal conductance of the leaf^50^.

### Floral traits: flower length

*Impatiens glandulifera* is known for its large, self-compatible, colourful flowers that produce copious quantities of nectar with the highest sugar content of any other annual species in Europe. This has been found to negatively affect native plants by being a successful competitor for pollinators^25^. This study found that an overall significant increase in the length of *I. glandulifera* flowers occurred between 1865 and 2017.

Although flowers of *I. glandulifera* are self-compatible, they are rarely self-pollinated and have a wide range of insect pollinators, mostly bumblebees (*Bombus* spp.) and honeybees (*Apis mellifera* L.)^51–53^. Flowers becoming longer may result in more deeply hidden nectar, which may benefit the plant by ensuring the nectar is not stolen by non-pollinating insects and in turn increasing pollen contact with the pollinator, hence increasing the efficiency of pollination^54^. In addition, assuming high correlation among floral traits^55^, it is plausible to speculate that an increase in flower length may correlate with an increase in flower size in *I. glandulifera*, which might provide an adaptive advantage in its non-native range, attracting a larger number and greater diversity of pollinators, as has been frequently observed in other species with large flowers^56–58^. In fact, pollinators have been shown to have a substantial effect on directional selection of flower size across different plant families, selecting for larger, more showy flowers that produce more nectar^57,59^. Possibly bumblebees and honeybees are selectively attracted to larger flowers and visit them more frequently; research has demonstrated that larger flowers are more easily seen by bumblebees along their foraging route compared to small flowers, and the bumblebees actively visit them more frequently^56^, which may result in an increased seed set^60^.

In this study we found a positive relationship between flower length and mean temperature, although we should be cautious interpreting this result due to the lack of support when re-running the models after under-sampling the original data. Elevated temperatures have been found to influence flowering traits, including pollen, nectar and flower production and also flower size^61^. However, no clear, consistent overall trends have been reported on the effects of warming on flower size, with some studies demonstrating a reduction, whilst others find an increase, pointing to species specific responses^61^. Regardless of whether the observed changes are driven by pollinators or due to increasing temperatures, it is clear that these changes may be capable of altering the interaction of *I. glandulifera* with pollinators.

## Conclusions

This study demonstrates the importance of herbarium collections for research into the dynamics of vegetation change^62,63^. Without the collections that were investigated here, it would have been impossible to provide the temporal / historical context for the evidence obtained from fieldwork. The work has revealed how *I. glandulifera* has undergone rapid phenotypic changes in the UK since 1865, in particular a reduction in leaf size and an increase in mean flower length. These morphological changes can be linked to an increase in competitive ability of the species and can be partly predicted under the EICA hypothesis^32^. The reduction in leaf size may be compensated for by an increased investment in reproductive structures. In addition, the longer *I. glandulifera* flowers may enhance the efficiency of pollination. The next logical step would be to look at how biotic interactions have been shaping this change; are there are other biotic (and abiotic) conditions, not addressed in this study, that may also be shaping flower evolution in *I. glandulifera*? Future increases in mean annual temperature associated with climate change may favour these trends and promote increasing flower length.

Adaptations of *I. glandulifera* that promote its spread and competitive success over native species have serious ecological and financial consequences, costing the UK government around £1,000,000 annually^23^. Understanding both the ecology of an invasive species, such as *I. glandulifera* and, the full range of its effects on the invaded ecosystem is essential in the formulation of effective control strategies and rehabilitation of invaded habitats^64^. The main methods of management include labour-intensive pulling, cutting, herbicide treatment with glyphosate or grazing^23^, while other studies have highlighted the effectiveness of using strains of rust originating from the native range of the species^29,65^. The evolution of *I. glandulifera* plants with smaller leaves may influence the choice of management technique, for example, by reducing the quantity of herbicide necessary for control.

The long-term consequences of the evolution of longer flowers are uncertain. *I. glandulifera* is favoured by beekeepers because it has an extended flowering time, coupled with high rates of sugar production, highlighting the potential use this species to support pollinating insects^66^. It has been suggested that the species has the potential to decrease genetic diversity in native plants as it lures pollinators away from natives^25^. In contrast, other research has suggested that an increase in species richness, visitor abundance and flower visitation was reported for plots invaded by *I. glandulifera* in multiple studies, resulting in a facilitated increase in pollinator visits for native species^64^. Further research is clearly needed to determine the impact of invasion by *I. glandulifera* on pollination of native species and to consider how this is affected by changes in adaptive traits highlighted by this research.

## Materials and methods

### Sampling herbarium specimens

**Figure 3:**
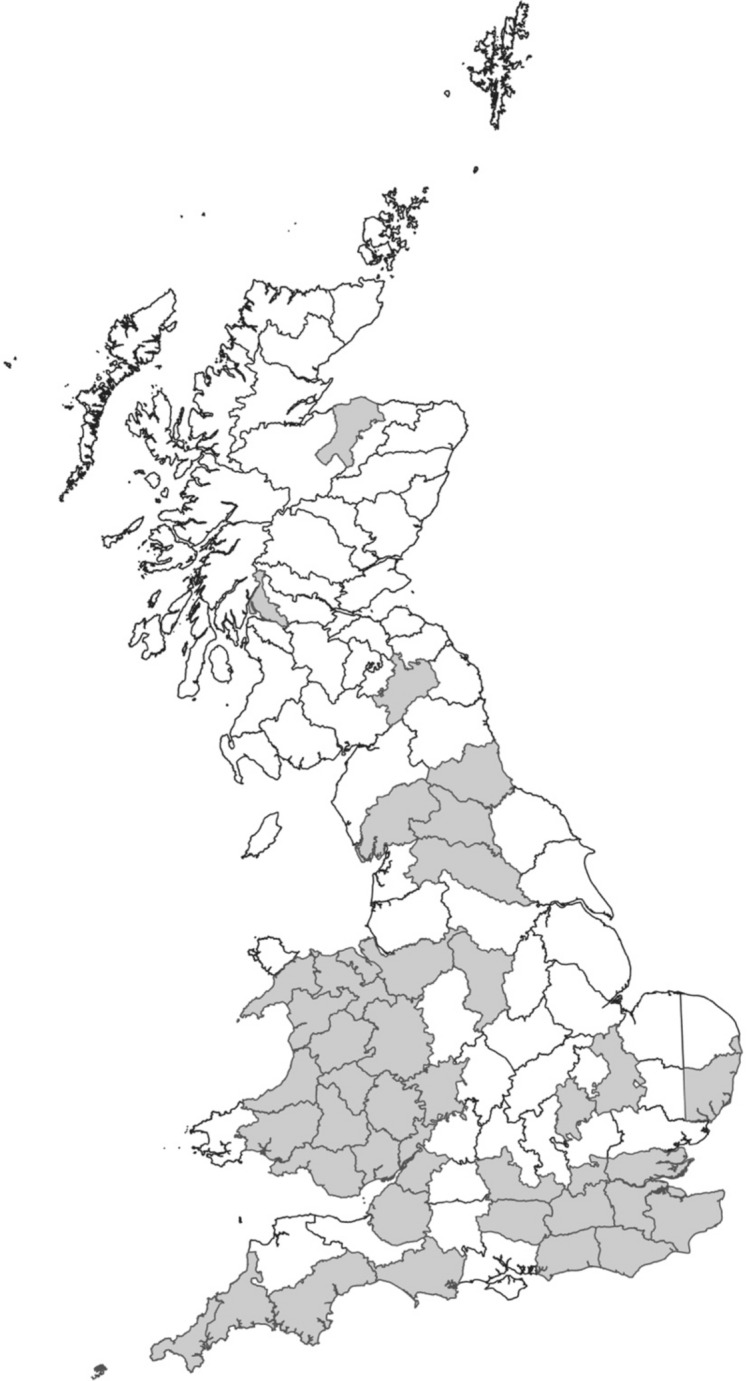
A GIS generated map showing the source of herbarium samples (i.e., vice-counties shaded in grey).

315 herbarium specimens were studied, with collection dates ranging from 1865 to 2017 (Tables S1 and S2, supplementary information). Specimens studied were located in the herbaria at Amgueddfa Cymru - Museum Wales, The Royal Botanic Garden Kew and the Natural History Museum London. Only high-quality specimens were sampled; any specimens that were badly damaged or had less than three fully intact leaves were excluded from the data analysis. Specimens were invariably from the apical portion of the plant. In this study, herbarium specimens were sampled from 41 vice-counties across the UK, covering most of Wales and southern England (Fig. 3).

### Field sampling

In 2017, data was obtained from 158 field specimens collected during June–July from 11 populations across South Wales (Table S3, supplementary information). Individuals were randomly selected from each habitat and each specimen was pressed and prepared using standard herbarium methods, hence imposing similar size reduction to historical specimens^67^. The specimens were collected from public areas, predominantly parks and waste ground. Where necessary, verbal permission was obtained by contacting park rangers and wardens. Specimens collected in the field have been deposited at the herbarium (NMW) of Amgueddfa Cymru - Museum Wales, where they are available for reference by researchers and members of the public. Individual voucher numbers are as followed V.2024.002.001–V.2024.002.159. The research project complied with legislation and guidelines for research issued by Cardiff University and the U.K.

### Morphological data

**Figure 4:**
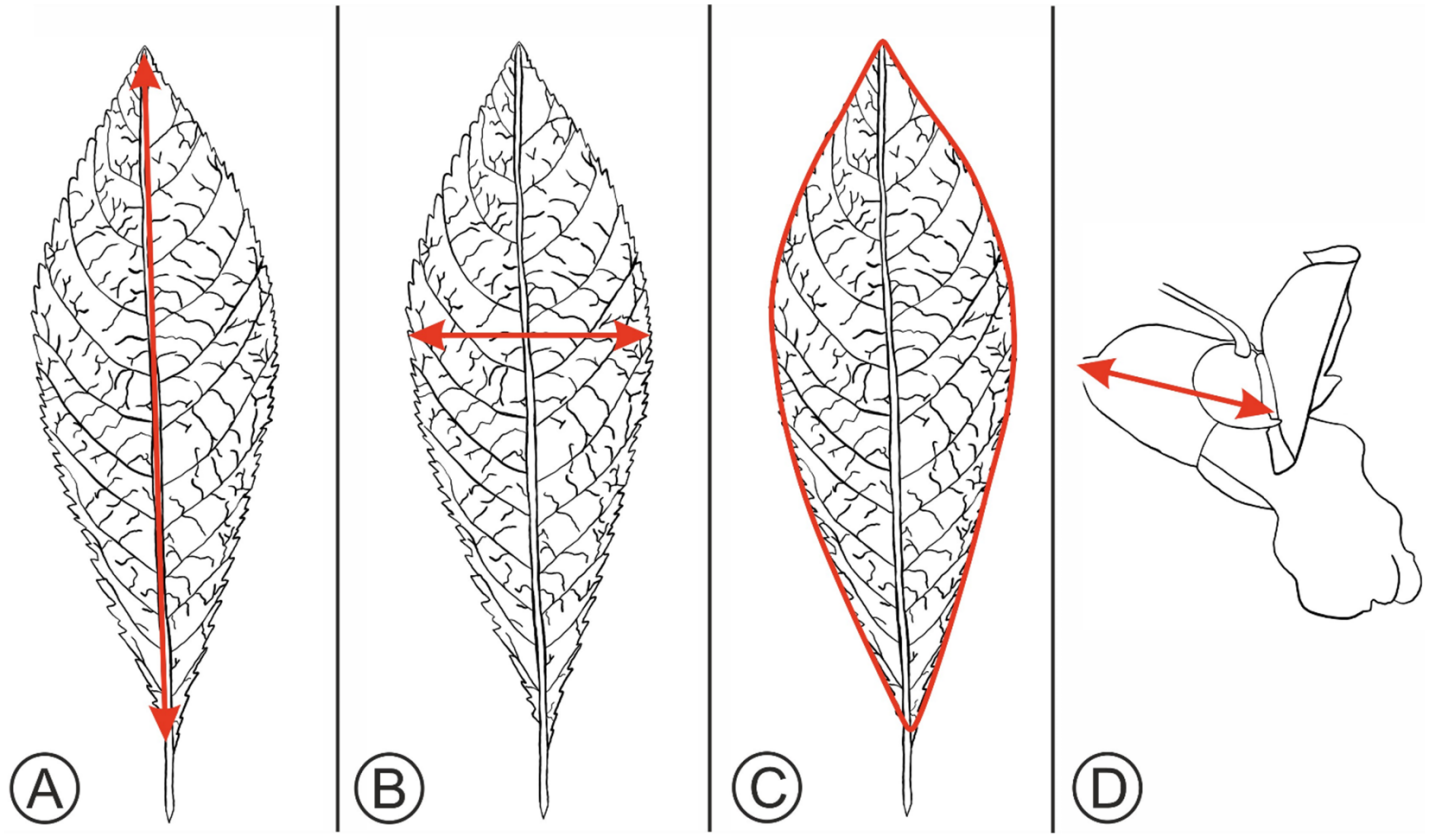
Morphology of *I. glandulifera* leaf and flower with illustrations of how each of the morphological measurements in this study were taken. (**A**) *I. glandulifera* leaf with a red line illustrating how the leaf length was calculated. (**B**) *I. glandulifera* leaf with a red line illustrating how the leaf width was calculated. (**C**) *I. glandulifera* leaf with a red line illustrating how the leaf area was calculated. (**D**) *I. glandulifera* flower with a red line illustrating how the length of the flower was calculated.

Digital photographs of each specimen were taken using a ruler for scale. Leaf area, length and width were all then calculated using Image J^68^. Any leaves that were located near the growing tip or that appeared immature were discounted from the analysis. Leaf length was calculated measuring from the tip of the apex to just before the petiole attachment (Fig. 4a), near the basal portion of the leaf. Leaf width was recorded as the widest portion of the leaf (Fig. 4b). The surface area of the leaf was measured as the circumference around the outside of the leaf (Fig. 4c). Where possible, five leaves were sampled per plant: where fewer leaves were available a minimum of three leaves were sampled.

Flower length was calculated by measuring the flower as shown in Fig. 4d. At least three flowers were sampled per plant.

Stomatal density was measured by coating the abaxial surface of the leaf with Germolene New Skin liquid plaster, leaving to dry for five to ten minutes and gently prying off the surface with a pair of fine tipped tweezers. This peel was then examined using a Nikon Labophot-2 Binocular Phase Contrast Microscope. The full procedure for this can be found at https://www.researchgate.net/publication/324784278_Non-invasive_method_for_looking_at_stomata_epidermal_cells_of_herbaria_specimens.

### Climatic data and geographic data

Mean monthly Central England Temperature (CET) records for the period 1865–2017 were obtained from the UK Meteorological Office (http://hadobs.metoffice.com/hadcet/cetml1659on.dat). These temperature series for Central England are representative of roughly a triangular area enclosed by Bristol, Lancashire and London^69^. It was found that monthly temperatures from stations distributed across the UK are highly correlated with the corresponding CET, indicating that its applicability extends beyond central England^70^. Longitude and latitude were calculated using the centroid of the Watsonian vice-county for each specimen.

### Statistical analyses

All statistical analyses were performed in R v. 4.2.2^71^. Multiple regression models were used to explore the relationship between traits measured (leaf area, leaf width, leaf length, flower length) and time, mean temperature of the growing season (mean of the monthly averages from March to November), latitude and longitude. As time was positively correlated with mean annual temperature (*Pearson’s r* > 0.7; Figure S1, supplementary information), different models were run for each of these two predictors to avoid issues due to multicollinearity. The package ‘visreg’ in R^72^ was used to allow the visualization of the relationship between the response variable (partial residuals) in relation to a given predictor while holding all other variables constant.

Models were validated via diagnostic plots of model residuals to verify the assumptions of normality and homoscedasticity^73^. Log10-transformation was applied to leaf area, length and width and to flower length to meet statistical assumptions. In addition, since the experimental data was highly unbalanced due to having a high number of samples for the last decade studied, we employed a random under-sampling approach limiting the data to 24 samples in the last decade. Random under-sampling was carried out 1000 times, and models were run on the new datasets. To assess potential biases in our original dataset, the percentage of models in which the *P*-value for each model coefficient was < 0.05 was calculated across the 1000 iterations.

### Supplementary Information

Supplementary Information.

## Data Availability

The data that support the findings of this study are available from the corresponding author, (ALW) upon request.
