# Supplementary material for: Rapid morphological change in UK populations of Impatiens glandulifera
Source: Sci Rep. 2024 Aug 20;14:19275. doi: 10.1038/s41598-024-69710-y (PMC11335755; doi:10.1038/s41598-024-69710-y)

**Supplementary information:**

Table S1. A table to show the number of herbarium and field collected specimens originating in different vice-counties

| **Specimens** | **Vice-County** |
| --- | --- |
| 1 | Bedfordshire |
| 1 | Berkshire |
| 2 | Breconshire |
| 4 | Caernarvonshire |
| 1 | Cambridgeshire |
| 1 | Cardiganshire |
| 1 | Carmarthenshire |
| 2 | Cheshire |
| 4 | Denbighshire |
| 3 | Derbyshire |
| 2 | Dorset |
| 2 | Dunbartonshire |
| 1 | Durham |
| 4 | East Cornwall |
| 1 | East Kent |
| 1 | East Suffolk |
| 1 | East Sussex |
| 1 | Flintshire |
| 99 | Glamorgan |
| 8 | Herefordshire |
| 7 | Merionethshire |
| 7 | Middlesex |
| 1 | Mid-West Yorkshire |
| 100 | Monmouthshire |
| 1 | Montgomeryshire |
| 2 | Moray |
| 1 | North Hampshire |
| 2 | North Somerset |
| 3 | North-West Yorkshire |
| 3 | Radnorshire |
| 1 | Roxburghshire |
| 1 | Shropshire |
| 14 | South Devon |
| 3 | South Essex |
| 16 | Surrey |
| 4 | West Cornwall |
| 3 | West Gloucestershire |
| 2 | West Kent |
| 1 | West Morland |
| 2 | West Sussex |
| 1 | Worcestershire |
| **315** | **TOTAL** |

Table S2. Specimen counts collected across various decades from 1860 to 2019.

| **Decade** | **Specimens** |
| --- | --- |
| 1860-1869 | 1 |
| 1890-1899 | 3 |
| 1900-1909 | 7 |
| 1910-1919 | 24 |
| 1920-1929 | 23 |
| 1930-1939 | 15 |
| 1940-1949 | 23 |
| 1950-1959 | 21 |
| 1960-1969 | 14 |
| 1970-1979 | 5 |
| 1980-1989 | 13 |
| 1990-1999 | 4 |
| 2000-2009 | 3 |
| 2010-2019 | 159 |
| **TOTAL** | **315** |

Table S3. Field collection locations, the number of collected specimens and the coordinates of the collection localities for specimens of *Impatiens glandulifera* collected in 2017.

| Locality name | Number of collected specimens | Coordinates |
| --- | --- | --- |
| Tredegar Park 1 – Ebbw river | 12 | 51°34'6.70"N, 3° 1'11.77"W |
| Tredegar Park 2- Towards Bassaleg | 20 | 51°34'23.43"N, 3° 2'14.78"W |
| Bettws lake | 10 | 51°36'18.31"N, 3° 0'41.30"W |
| Bute Park 1 | 20 | 51°29'11.08"N, 3°11'17.68"W |
| Bute Park 2 | 20 | 51°29'4.86"N, 3°11'6.19"W |
| Bute Park 3 | 10 | 51°28'59.99"N, 3°10'57.38"W |
| Caerleon - Usk Road | 20 | 51°36'54.73"N, 2°57'2.14"W |
| Llantarnam Lake | 8 | 51°38'24.45"N, 3° 0'22.55"W |
| Roath Park | 20 | 51°30'14.24"N, 3°10'27.11"W |
| Taff River - Pontcanna | 9 | 51°29'47.19"N, 3°12'26.86"W |
| Taff River - Taly | 9 | 51°29'44.12"N, 3°11'42.12"W |

Figure S1. Correlation matrix plot displaying the Pearson correlation coefficients between paired explanatory variables: Year of collection, longitude, latitude and mean average temperature for the period of March to November (T9months) for each of the samples of *I. glandulifera* used in the study. The bivariate scatter plots with a fitted red line are presented on the bottom of the diagonal, the values of the correlations are displayed. on the top of the diagonal. This plot was generated by using the function ‘pairs’ in R program.


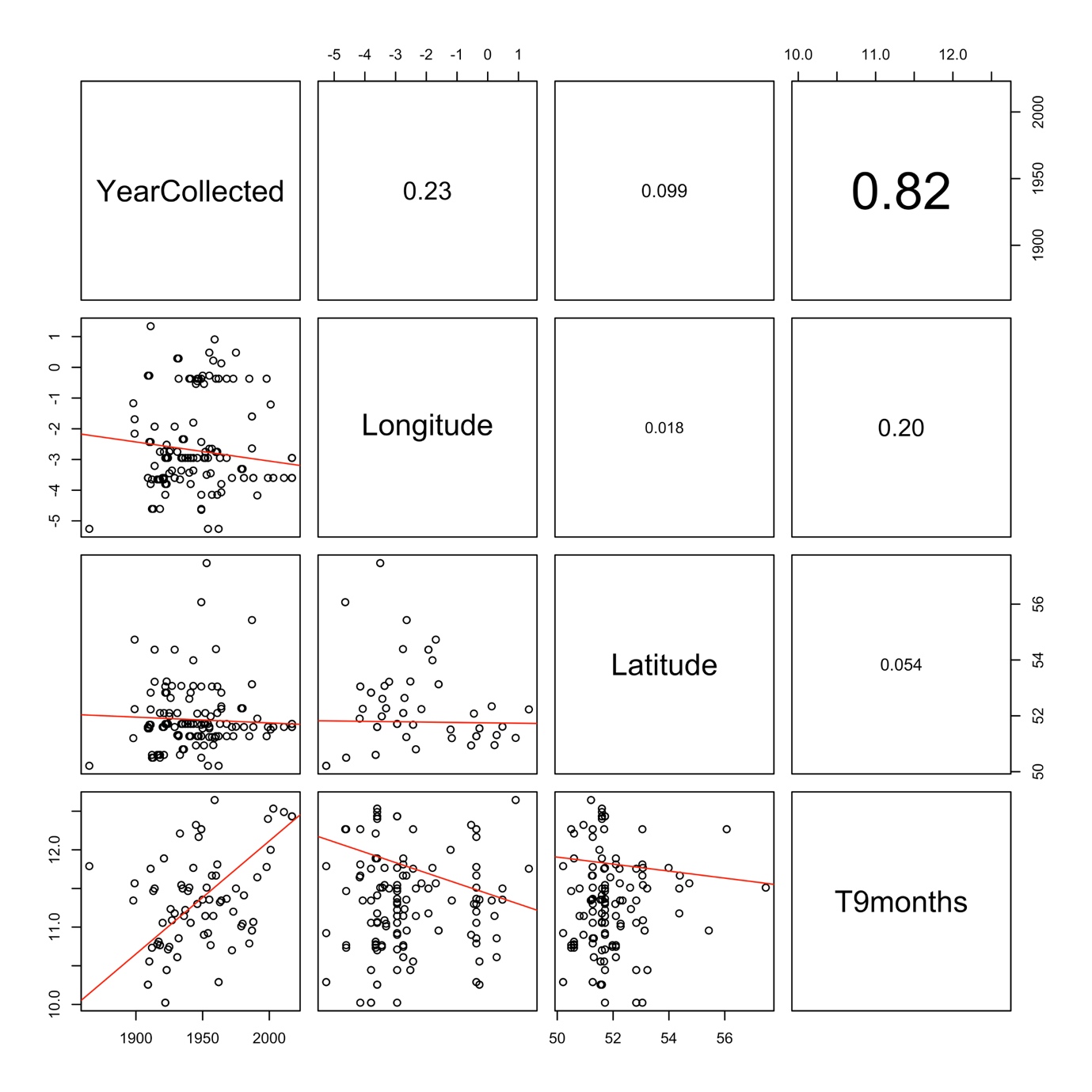

Supplement: Supplementary file 1 — Supplementary Information. [file 41598_2024_69710_MOESM1_ESM.docx]
